# Factors Associated with Postsurgical Pain and Swelling Following Endodontic Microsurgery: The Role of Radiographic Characteristics

**DOI:** 10.3390/healthcare13090995

**Published:** 2025-04-25

**Authors:** Abdulwahed Alghamdi, Dana Mominkhan, Reem Sabano, Noha F. Alqadi, Mey Al-Habib, Sarah Bukhari, Mohammed Howait, Loai Alsofi

**Affiliations:** 1 Department of Endodontics, University Dental Hospital, King Abdulaziz University, P.O. Box 80209, Jeddah 21589, Saudi Arabia; amaalghamdi7@kau.edu.sa (A.A.); rsabano@kau.edu.sa (R.S.); 2 Department of Endodontics, School of Dental Medicine, University of Pennsylvania, Philadelphia, PA 19104, USA; mominkhan@uthscsa.edu; 3 Department of Endodontics, Faculty of Dentistry, King Abdulaziz University, P.O. Box 80209, Jeddah 21589, Saudi Arabia; nfalqadi@kau.edu.sa (N.F.A.); maalhabib@kau.edu.sa (M.A.-H.); smnbukary@kau.edu.sa (S.B.); mhowait@kau.edu.sa (M.H.)

**Keywords:** endodontic microsurgery, pain, edema, predictors, cone beam computed tomography, questionnaire

## Abstract

**Objectives**: Endodontic microsurgery has become an integral part of daily endodontic practice. However, research on the correlation between the lesion characteristics observed via cone beam computed tomography (CBCT) and pain and swelling after endodontic microsurgery (EMS) is still lacking in the literature. The present study aims to examine the relationship between the radiographic characteristics of preoperative periapical lesions obtained from CBCT images and post-surgical symptoms such as pain and swelling. **Materials and Methods**: A total of 61 patients undergoing EMS utilizing modern techniques were asked to report their level of pain and swelling at 8, 24, 48, and 72 h after EMS using VAS. Independent variables such as age, gender, tooth location, CBCT periapical index, endodontic diagnosis, cortical bone perforation by the lesion, duration of the EMS, preoperative analgesic consumption, antibiotic prescription, and pre-/postoperative mouthwash were analyzed using the Fisher Exact test. Multivariate regression analysis was also conducted to determine the independent significant factors associated with pain and swelling. A *p*-value of ≤0.05 was considered statistically significant. **Results**: The maximum pain score was recorded at 8 h (4.26 ± 3.13), while peak swelling was measured after 24 h (6.46 ± 2.87). The risk of swelling was more likely to decrease by 75.7% for patients with a CBCT index score of >3 than those with a CBCT index score of ≤3 (AOR = 0.243; CI = 0.071–0.831; *p* = 0.024). The effects of all other factors on pain, including cortical bone perforation by the lesion (*p* = 0.290), swelling (*p* = 0.071), postoperative mouthwash use (*p* = 0.062), and swelling (*p* = 0.934), did not reach statistical significance. **Conclusions:** Patients with periapical lesions larger than 4 mm will likely experience less swelling after EMS, while pain is not affected by lesion size, cortical bone perforation, or mouthwash use. **Clinical Relevance**: This study identified a new predictor of swelling after EMS based on the size of the periapical lesion. These results will improve the management of post-surgical sequelae after EMS and support shared decision making.

## 1. Introduction

Apical periodontitis is an inflammatory disease that affects the tooth and its supporting apparatus, the periodontal ligament, and the surrounding alveolar bone. Nonsurgical root canal treatment for teeth with apical periodontitis shows favorable outcomes and root canal-treated teeth have a high survival rate [1,2]. To achieve this successful outcome, treatment should aim to eradicate the infected inner dentin layer to allow for the physical disruption of the bacterial biofilm, complete debridement, and the disinfection of the radicular space, followed by fluid-tight seal obturation [1,3,4]. However, periapical lesions may persist after nonsurgical endodontic treatment and nonsurgical retreatment. This can be attributed to the persistent or secondary intraradicular infection, the presence of extraradicular infection, foreign body reaction, or the presence of a cystic lesion [5]. The persistence of apical periodontitis necessitates surgical intervention to eradicate the infection, enucleate the lesion, and fill the root canal end with a biocompatible material.

The primary objectives of any surgical procedure are to effectively manage acute pain during the preoperative period, support the patient’s psychological well-being, and improve their overall quality of life, particularly for those experiencing immediate postoperative discomfort [6,7,8,9]. Periapical surgery is a specialized therapeutic procedure designed to address periapical inflammation in teeth, especially in cases where orthograde retreatment is challenging or unsuccessful in resolving the apical pathology [10,11]. Endodontic microsurgery (EMS) has gained recognition as a predictable treatment in recent years. Advancements such as the use of operating microscopes and microsurgical techniques have significantly improved outcomes, with success rates for apical periodontitis reaching up to 94%, compared to around 60% with traditional root-end surgery [11,12,13,14,15,16,17,18]. EMS is now an integral part of routine endodontic practice.

Like any surgical procedure, periapical surgery results in tissue damage, which can lead to both local and systemic effects that can negatively impact a patient’s quality of life. Immediate postoperative outcomes, such as pain, swelling, and the patient’s overall well-being after periapical surgery, have received limited attention historically. However, there is a growing number of studies focusing on the impact of endodontic surgery on quality of life during the recovery period [5,19,20,21,22,23,24,25]. In general, pain and swelling following oral surgical procedures have been discussed intensively in the literature with pain being the most common postoperative complication after endodontic microsurgery [25,26,27,28]. Postoperative symptoms depend mainly on the difficulty of the procedure. Postoperative pain reaches its maximum level during the first 7 days, with maximum intensity recorded in the first 48 h, and is short in duration [29]. Patient factors such as age and sex, along with tooth-related factors such as position, may increase the risk of postoperative pain [25,26]. Flap design, the duration of surgery, and the number of teeth treated have also been linked to postoperative inflammation, pain, and swelling [27]. Penarrocha et al. have found that poor oral hygiene contributes to postoperative pain and increases analgesic consumption [30]. Compared to the nonsurgical approach for treating endodontic diseases and other oral surgical procedures, the factors that influence the post-surgical sequelae after EMS are lacking in the literature.

With recent advances in technology, dental radiology has seen the introduction of several new imaging techniques that are transforming diagnostics. Tools such as digital X-rays, densitometry, cone beam computed tomography (CBCT), MRI, ultrasound, and nuclear imaging now offer incredibly detailed, high-resolution views of oral structures [31,32]. These innovations make it possible to detect bone lesions and other issues earlier than ever, improving both diagnosis and patient care. With the growing use of cone beam volumetric tomography, its specific applications in endodontics are becoming clearer [33]. Several studies highlight potential uses, including diagnosing conditions of both endodontic and nonendodontic origins, analyzing root canal morphology, evaluating root and alveolar fractures, assessing external and internal root resorption, identifying invasive cervical resorption, and aiding presurgical planning for root-end surgeries [33,34,35,36]. Scarfe et al. emphasized that key innovations in imaging include the shift from analog to digital systems and advancements in imaging theory and 3D volume acquisition, which provide highly detailed imaging [37]. CBCT has also been used to distinguish between endodontic and nonendodontic lesions, aiding treatment planning [33]. CBCT offers superior resolution, faster scans, and lower radiation. As it becomes a standard tool, it reduces reliance on guesswork, improving outcomes for both patients and clinicians. Its full potential—from diagnosis to image-guided procedures—remains to be fully explored [38].

Despite its growing popularity and widespread adoption in daily endodontic practice, the past decade has seen limited research on postoperative pain and swelling following EMS. Moreover, no published research assessing the relationship between periapical lesion size and cortical bone erosion and post-surgical pain and swelling after EMS is available. This study aims to examine the relationship between the periapical lesion characteristics observed from CBCT scans, namely size and cortical bone perforation, in patients undergoing EMS and postoperative symptoms such as pain and swelling.

## 2. Compliance with Ethical Standards

### 2.1. Ethical Approval

The Research Ethics Committee at the Faculty of Dentistry, King Abdulaziz University, approved this research before its commencement (ethical approval number: 18-01-18).

### 2.2. Informed Consent

Written consent was obtained from all patients before performing the surgical procedure and for participation in this study.

## 3. Materials and Methods

Patients attending postgraduate endodontic clinics for EMS were included in this study after giving consent. Patients aged 18 years or older with periapical lesions requiring EMS via surgical access from the facial aspect were included in this study. Exclusion criteria encompassed individuals with significant cognitive impairments, an inability to complete the necessary questionnaires in Arabic, diabetes, immune disorders, or cardiovascular conditions restricting the use of higher doses of epinephrine. Additionally, any recent illness requiring antibiotic or analgesic treatment were excluded. Additionally, patients who reported recent illnesses requiring antibiotic or analgesic treatment were excluded.

Before the planned day of EMS surgery, the patients were interviewed and handed their questionnaires. On the day of the interview, demographic data were collected, such as age, gender, and ethnicity. The interviewer explained the questionnaires and how to fill them out to the patients. To ensure accurate self-assessment of facial swelling using the Visual Analog Scale (VAS), subjects underwent structured training. Initially, they were introduced to the concept of facial swelling assessment, including its clinical significance and the rationale behind using the VAS. Facial swelling was defined as the presence of puffiness or enlargement in specific regions of the face, such as around the eyes, cheeks, or jawline. The severity of facial swelling was characterized by variations in the extent and prominence of swelling, which could range from mild puffiness to more pronounced and noticeable enlargement. The scale, typically a 10 cm horizontal line with endpoints labeled “No swelling” (score 0) and “Maximum swelling” (score 10), was clearly explained, and subjects were familiarized with its interpretation. Swelling was scored according to the criteria established by Garcia et al. as follows: 0, or “none”, indicated no observable swelling; 1–3, or “mild”, referred to intraoral swelling confined to the surgical field without noted facial asymmetry; 4–6, or “moderate”, described extraoral swelling limited to the surgical area; and 6–10, or “intense”, denoted extraoral swelling extending beyond the surgical area such as around the eyes [39]. Demonstrations were provided, explaining how to observe swelling in a well-lit mirror, evaluate its severity, and mark the corresponding point on the scale. Emphasis was placed on the importance of standardized conditions, such as assessing swelling at the same time each day under similar lighting conditions. Additionally, potential challenges, such as subjective variability, were addressed by reinforcing the need for honest and precise self-reporting. To enhance long-term accuracy, subjects were encouraged to document their assessments systematically and track changes over time, with one of the investigators providing periodic feedback to ensure methodological adherence. Patients were instructed to take analgesics every 8 h, specifically at 8, 16, 24, 32, 40, 48, 56, 64, and 72 h post-surgery. They were also directed to record their levels of pain and swelling at 8, 24, and 72 h post-surgery [24]. Importantly, patients were advised not to delay recording their responses to account for the onset of analgesic effects but to adhere strictly to the prescribed time points for documentation.

**Figure 1:**
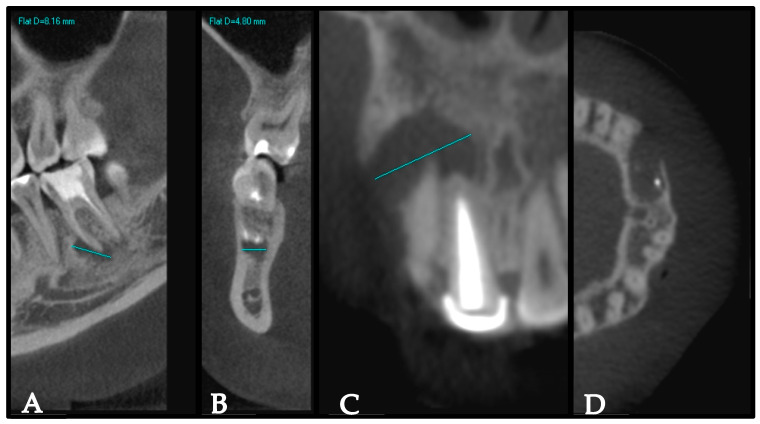
Representative samples illustrating measurements of the periapical lesion and cortical bone perforation using the CBCT images. (**A**,**C**) represent the largest extension of the periapical lesion. (**B**) represents the intact cortical bone on the coronal view in tooth #46. (**D**) represents Axial view showing erosion of the labial cortical bone by the lesion.

As part of EMS planning, CBCT scans of the surgical area (maxilla, mandible, or both) were obtained for all participants using an i-CAT CBCT machine (Imaging Sciences International, Inc., Hatfield, PA, USA). The field of view was 6 cm with a 0.4 mm voxel size, and the acquisition time was 20 s. The periapical lesions were scored based on the periapical index on CBCT [40]. In brief, the largest extension of the periapical lesion was recorded on either the buccolingual, mesiodistal, or diagonal dimensions. Then, the periapical index was determined as a score of 0 if the periapical bone was intact, a score of 1 with a lesion size of 0.5–1 mm, a score of 2 with a lesion size of 1–2 mm, a score of 3 with a lesion size of 2–4 mm, a score of 4 with a lesion size 4–8 mm, and a score of 5 with a lesion size >8 mm. In instances where multiple non-fused periapical lesions were present on a single tooth, the CBCT periapical index was classified according to the dimensions of the largest periapical lesion associated with that tooth. This approach ensured that the most significant lesion determined the overall categorization, providing a standardized method for assessment. Cortical bone destruction or expansion was recorded as well. The intraexaminer reliability was evaluated by conducting repeated measurements on 10% of the sample after a one-week interval. A 92% agreement rate and an intra-class correlation coefficient (ICC) of 0.85 were achieved, demonstrating strong reliability. Figure 1 shows representative samples illustrating the measurements of the periapical lesion and cortical bone perforation using the CBCT images.

Chlorhexidine mouthwash 0.12% was prescribed twice daily for two days preoperatively [41], and a single dose of 400 mg of ibuprofen was provided 1 h before EMS [24], which is also in accordance with the department’s protocol.

All surgical procedures were performed by endodontic residents and specialists, utilizing modern microsurgical endodontic techniques, who were blinded to the objectives of the ongoing research. Local anesthesia was administered using 2% Lidocaine with 1:50,000 epinephrine for infiltration and an inferior alveolar nerve block. All surgical procedures were carried out using a dental operating microscope (OPMI PICO, Carl Zeiss, Oberkochen, Germany), including flap elevation, osteotomy, curettage, root-end resection, preparation, inspection, and filling. Racellets (Pascal Co., Bellevue, WA, USA) were used as an adjunct hemostatic agent inside the bony crypt. Root-end preparation was performed using ultrasonic tips (Acteon, Endo-Tech, Halifax, NS, Canada), and root-end preparation was filled with either ProRoot MTA (MTA; Dentsply Tulsa Dental Specialties, Memphis, TN, USA) or TotalFill BC RRM putty (FKG Dentaire SA, La Chaux-de-Fonds, Switzerland). After flap repositioning, a postoperative periapical film was acquired to check the levels of root resection, preparation, and filling before wound closure, followed by flap suturing.

As a general protocol in the department and for standardization purposes, analgesics were prescribed to all patients [41]. Antibiotics were prescribed selectively for patients with severe symptoms associated with acute infections [41].

Patients were instructed to take one pill of 400 mg of ibuprofen an hour before EMS and continue taking it three times daily after surgery (specifically at 8, 16, 24, 32, 40, 48, 56, 64, and 72 h post-surgery) [24]. We selected three days post-surgery as the observation period because it is well-documented in the studied population that there is a high dropout rate. Therefore, three days was deemed the most suitable timeframe to optimize the response rate, particularly given the small sample size.

If the pain was unbearable, patients were instructed to take 500 mg of acetaminophen along with ibuprofen. Post-surgical procedure instructions were given, with the patients instructed to use ice packs on and off for twenty-minute intervals for the following eight hours [42,43]. Patients returned for suture removal and follow-up three to four days following EMS. On that day, the questionnaires were collected, and the patients were interviewed briefly about their experience. The patients’ self-reported levels of pain and swelling questionnaires consisted of two parts, as previously reported [26]. The first part included a Visual Analog Scale (VAS) for the patients to record their pain occurrence after EMS at 8, 24, 48, and 72 h (0 = no pain; 10 = severe pain). The second part consisted of a similar scale to record the patient’s experienced swelling at the same time intervals. The patients were instructed to report their subjective swelling experience on the VAS, where a score of 0 means no swelling and a score of 10 means severe swelling.

## 4. Statistical Analysis

All data analyses were performed using the statistical package for social sciences, version 21 (SPSS, IBM Corp, Armonk, NY, USA). Descriptive statistics were presented as counts, proportions, means, and standard deviations whenever appropriate. The relationship between pain and swelling among the baseline characteristics of patients was conducted using the Fisher Exact test. A *p*-value of ≤0.05 was considered statistically significant. Multivariate regression analysis was also performed to determine the independent significant factors associated with pain and swelling. The overall mean scores of both pain and swelling were generated by calculating the mean scores of four different time points measured at 8 h, 24 h, 48 h, and 72 h.

## 5. Results

Sixty-one patients who underwent EMS were analyzed. Table 1 presents the baseline characteristics of the analyzed patients. The mean age was 39.8 years, with nearly two-thirds of the patients (65.6%) being in the younger age group (≤40 years). Approximately 90% of participants had no chronic diseases, while 9.8% were classified with chronic illnesses—all of whom were female patients with well-controlled hypothyroidism. A minority (14.8%) reported being smokers. More than 77% had one tooth operated on, with 37.3% of those teeth located in the posterior maxilla and 33.9% located in the anterior maxilla.

With regard to the CBCT index score, the majority had more than a three-point score (61%), and a high proportion had cortical bone perforation on CBCT (61%). We further observed that more than half of the participants were symptomatic at the time of EMS (52.5%), and 18% had sinus tracts. Upon flap elevation, 54.1% had clinical cortical bone perforation. The mean duration of operation was 131.6 min (SD 50.5); however, more than half of the operations lasted less than 2 h. Moreover, approximately 63% of the participants used the prescribed analgesics and about 60% used the prescribed mouthwash preoperatively. For the postoperative characteristics of patients regarding the prescribed mouthwash and analgesics, 93.4% used the mouthwash, and 67.2% took either analgesics or antibiotics.

**Figure 2:**
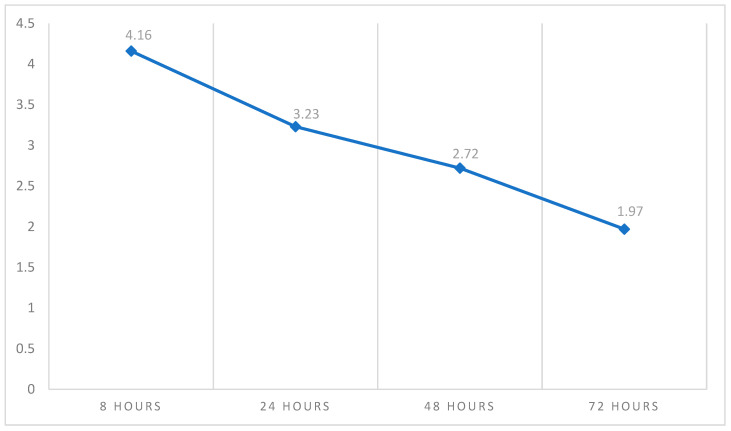
The trendline average of pain measured over time. This figure shows that the peak of postoperative pain occurs at 8 h, before declining.

**Figure 3:**
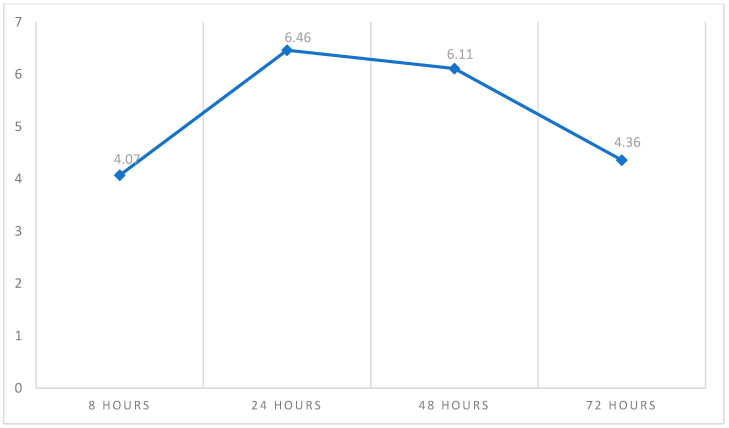
The trendline average of swelling measured over time. This figure shows that the peak of postoperative swelling occurred after 24 h and then declined.

Table 2 shows the descriptive statistics of pain and swelling measured over time on four different occasions. The pain score was higher at 8 h (4.26 ± 3.13) and relatively lower after 72 h (1.97 ± 2.44). On the other hand, the overall mean pain score was 3.04 ± 2.18, as shown in Figure 2. With regard to swelling, Figure 3 shows that the peak of swelling was measured after 24 h (6.46 ± 2.87), while it was lower after 8 h (4.07 ± 2.91). The overall mean swelling score was 5.25 ± 2.18.

When assessing the relationship between overall pain and swelling among the baseline characteristics of patients, taking mouthwash preoperatively was significantly associated with increased pain (*p* = 0.048). With regard to the swelling level postoperatively, those with a CBCT index of more than three points (*p* = 0.029) and those with cortical bone perforation (*p* = 0.040) were significantly less associated with swelling, while those using mouthwash postoperatively (*p* = 0.020) were significantly more associated with swelling.

On the other hand, other variables included in this study, namely age in years, gender, medical history, smoking, teeth count, tooth location, CBCT cortical bone perforation, clinical presentation, sinus tract, duration in minutes, preoperative analgesics, postoperative antibiotics, and postoperative analgesics, were not statistically significantly associated with the pain or swelling incidence level, as shown in Table 3.

When conducting multivariate regression analysis estimates to predict the independent risk factors associated with pain and swelling, the odds of swelling for those patients with more than a score of 3 in the CBCT index were more likely to decrease by 75.7% than for those with a CBCT index of 3 or less (AOR = 0.243; CI = 0.071–0.831; *p* = 0.024). In contrast, intraoperative cortical bone perforation and preoperative and postoperative mouthwash did not reach statistical significance in association with either pain or swelling after adjustment to the regression model, as shown in Table 4.

## 6. Discussion

Patient preferences and expectations are central to the planning of surgical procedures [25,44,45,46]. Postoperative pain and swelling, common after endodontic surgery, often reflect tissue damage and can impact recovery and quality of life [26,27,28,47,48]. Identifying patient- or treatment-related risk factors for severe pain and swelling can improve management, enhance comfort, and guide decision making [48]. This study aimed to assess the relationship between preoperative periapical lesion characteristics and postoperative pain and swelling following EMS. Although previous research has linked factors such as age, sex, tooth type, osteotomy size, flap design, procedure duration, and use of a dental operating microscope to pain levels, most studies are over a decade old [26,27,28,42]. Recent innovations, including the use of CBCT for precise preoperative planning and the introduction of premixed tricalcium silicate-based root-end filling materials, have shortened surgery times and improved outcomes [48].

In our study, pain and swelling were patient-reported outcome measures assessed over three days only to ensure patient compliance and increase the response rate. Moreover, it has been shown that the highest pain and swelling intensities occur in the first three days after surgery [26]. The CBCT periapical index was used to quantify the characteristics of the periapical lesion before EMS. This index has been validated previously and is widely used in the endodontic literature [40]. The accuracy of CBCT machines in detecting cortical bone perforation was questioned previously and shown to be high [49]. However, this assumption was not observed in our results because of the limitations of large-field-of-view CBCT and large voxel size.

Our results show that pain after EMS is not severe. The prescription of pre-operative ibuprofen was not due to pre-existing pain complaints but rather based on pharmacological rationale. First, the local anesthetic effect typically persists for 1–2 h postoperatively, coinciding with the peak release of key pain mediators. A single 400 mg oral dose of ibuprofen reaches peak plasma concentration within 1–2 h, maintaining therapeutic tissue levels for approximately 2–6 h—thus ensuring adequate analgesia before the local anesthetic subsides. Second, ibuprofen inhibits prostaglandin synthesis rather than neutralizing or blocking preformed prostaglandins. The administration of pre-operative ibuprofen may have contributed to the non-significant postoperative pain outcomes observed in this study since 38 patients adhered to the prescribed analgesics. According to the best available evidence on pain after oral surgical procedures, preoperative and postoperative ibuprofen and acetaminophen prescriptions were shown to be effective in pain control [50]. Thus, we decided to follow that approach in our study instead of issuing as-needed prescriptions.

In this study, we assessed how endodontic microsurgery can affect postoperative pain and swelling. The maximum pain score was reported at 8 h and decreased constantly until it reached its lowest score after 72 h. This finding was in agreement with previous research that showed maximum pain intensity on the day of EMS [27,48,51,52]. The results of VAS show that pain intensity was highest at 8 h after surgery (4.26 ± 3.13), before decreasing at 24 h (3.23 ± 2.55), 48 h (2.72 ± 2.55), and 72 h (1.97 ± 2.44). These results are consistent with previous reports. A study by Tuk et al., 2021, evaluated the effects of apical surgery on oral health-related quality of life in the first postoperative week [24]. The results showed that the mean numeric rating scale of pain score was highest during the first three days, with a score of 3.25 (SD 2.47) on day 1, decreasing to 2.57 (SD 2.42) on day 2 and continuing to decline gradually over the week [24]. Iqbal et al. reported a mean NRS pain score of 3.17 (SD 2.03) on the first postoperative day [26]. Garcia et al. reported the highest pain score on the second postoperative day [39]. Several other reports investigated the occurrence of pain in 7-day periods postoperatively, and patients experienced the most severe pain on the day of surgery [53].

Swelling is a common occurrence following surgical periapical treatment. In our study, a swelling score of 4.07 ± 2.91 was recorded 8 h after surgery. Swelling; however, peaked at 24 h (6.46 ± 2.87) and 48 h (6.11 ± 2.79) after surgery. After 72 h, swelling gradually improved and the score came down to 4.36 ± 2.77. This was in agreement with previous reports [26,27,41]. In a study by Tuk et al., a significant difference in postoperative swelling was observed between genders on days 1 and 4, with women reporting more swelling than men. Additionally, swelling persisted longer in mandibular sites, showing significant differences on days 5, 6, and 7 [24]. Notably, patients were more likely to experience swelling than pain, supporting previous reports that identify the first postoperative day as the peak day for swelling [26].

In our study, factors such as age in years, gender, medical history, smoking, teeth count, tooth location, CBCT cortical bone perforation, clinical presentation, sinus tract, duration in minutes, preoperative analgesics, postoperative antibiotic, and postoperative analgesics were not statistically significantly associated with pain or swelling. In our study, only six patients were classified as having chronic illnesses, all of whom were female with well-controlled hypothyroidism. To the best of our knowledge, existing literature does not report any significant influence of hypothyroidism on post-surgical pain and swelling. Previous reports show that age and sex were linked to the levels of pain and swelling after EMS [26]. Females and young patients were shown to have lower pain thresholds and reported more pain [26]. There is controversy in the literature regarding the correlation between age and pain and swelling occurrence. In a previous report, patients under 25 experienced a greater overall impact from periapical surgery during the first two days, with higher pain levels reported on the first postoperative day [24]. However, other studies have not found age to be a significant factor influencing postoperative symptoms following periapical surgery, suggesting that age alone may not consistently predict patient outcomes [39,41,42,54]. On the other hand, Iqbal et al. found increased postoperative discomfort in younger patients [26]. In our results, age and gender did not influence pain and swelling. This could be attributed to the smaller sample size studied. As for gender variability, several studies reported no significant differences in pain scores between males and females following apical surgery [41,42]. Concerning smoking, Garcia et al. and Tuk et al. reported that smokers experienced greater pain during nearly the entire first postoperative week [24,39]. Several other reports did not show correlations between smoking and postoperative pain and swelling [24,39]. 

In this study, the average surgical time was 120 min. Past studies have reported wide variability in operation times, ranging from an average of 25 min to as long as 140 min for single-rooted teeth. However, no significant correlation has been found between operation time and postoperative pain or swelling [20,27,28]. Tooth location did not affect the occurrence of postoperative pain or swelling, which is consistent with previous reports [20,27,28,41,42]. Other studies have reported greater postoperative pain following periapical surgery on maxillary anterior teeth [26], molars [9,54], and lower incisors and canines [27]. The use of modern surgical techniques and modern reprofiling materials such as MTA and bioceramic materials help with reducing surgical time and improving treatment outcomes [55,56,57]. A detailed meta-analysis by von Arx et al. explored potential prognostic factors affecting healing outcomes in apical surgery [58]. Their findings indicated more favorable outcomes in cases lacking preoperative pain or symptoms, those with well-compacted root canal fillings, and those where periapical lesions were absent or smaller than 5 mm. Although the study evaluated several patient- and treatment-related variables, including age, gender, tooth location, dental arch, the size of the periapical radiolucency, and history of retreatment, none were found to significantly influence the surgical outcome [58].

Multivariate regression analysis was used to adjust for confounders and quantify the independent effect of CBCT index and intraoperative cortical bone perforation association with swelling, preoperative mouthwash association with pain, and postoperative mouthwash with swelling. The odds of swelling for those patients with more than a score of 3 in the CBCT index were more likely to decrease by 75.7% than for those with a CBCT index of 3 or less (AOR = 0.243; CI = 0.071–0.831; *p* = 0.024). In contrast, intraoperative cortical bone perforation, preoperative mouthwash, and postoperative mouthwash did not reach statistical significance. Studies show that the use of chlorohexidine mouthwash helps in reducing the recoverable bacterial flora from the surgical sites, thus reducing the possibility of postoperative infection, hence improving the healing process [41]. The advantages of preoperative analgesia have already been discussed, and when implemented, this approach should help to minimize the need for postoperative pain management [59]. The use of preoperative mouthwash and analgesics is necessary to standardize all factors that could impact the occurrence and intensity of postoperative symptoms, thus enabling accurate analyses of the variables under study. In our study, preoperative analgesics were prescribed to all patients regardless of the severity of pre-excising symptoms and only 38 patients took the medication.

At the same time, only the CBCT periapical index score was associated with more swelling after conducting the multivariate regression analysis estimates. When it comes to cortical bone perforation, it is not surprising that lesions involving the destruction of both cortical plates (“through and through” lesions) had poorer healing (75%) compared to those with no cortical plate destruction (87%) or the destruction of only one plate (91.84%) [60]. However, no correlation was found between cortical bone perforation and healing one year after surgery [61]. The risk of swelling for patients with more than a 3-point score in the CBCT index was more likely to decrease by 75.7% than those with a CBCT index score of 3 points or less. For CBCT periapical index score of 3, the lesion dimension was 2–4 mm, which meant that the clinician had to remove more bone when performing the osteotomy. Patients with large periapical lesions that had eroded the cortical bone exhibited chronic inflammation resulting from persistent microbial infections. This chronic inflammatory process was characterized by the continuous release of pro-inflammatory mediators such as histamine, bradykinin, and prostaglandins at the local site [62]. The additional elevation of these mediators due to surgical trauma may be limited and does not have the same impact as it would if healthy, intact bone needed to be removed to access the tooth apex, which may explain the lower risk of swelling in these patients. When it comes to postoperative pain, larger or more complex lesions, such as those involving multiple cortical plates, were associated with higher pain levels [63]. However, in the present study, no significant factors were found to influence postoperative pain levels. This lack of association may be attributed to the limited sample size.

The lack of a control group is one of the limitations of this study. Ideally, a negative control group would consist of patients with healthy, normal periapical tissues. However, such patients would not be indicated for endodontic microsurgery. We acknowledge that this inherent limitation in the study design cannot be resolved, which is why we adopted a prospective cohort design. Another limitation of our study that should be considered when interpreting the results is the relatively small sample size, which reduces the statistical power of our analyses and increases the risk of Type II errors. This may compromise the generalizability of our findings and could have affected the statistical analysis of some factors, such as cortical bone perforation by the lesion and postoperative pain. Cortical bone thickness was not assessed in this study due to the limited accuracy associated with large FOV scans and large voxel sizes. However, Malagise et al. reported that the likelihood of experiencing severe pain after EMS increased by 1.4 times for every 1 mm increase in cortical bone thickness [48]. Surgical trauma to the biological tissue induces edema, pain, and trismus due to the expression of endogenous inflammatory mediators such as prostaglandin, prostacyclin, histamine, and bradykinin [64]. Moreover, the heat generated during bone removal in surgery can trigger significant inflammation at the surgical site. This inflammation often leads to increased postoperative pain and swelling [65]. This is different from our results as, in our study, the majority of patients had cortical bone perforation.

Another limitation is that CBCT has notable limitations in detecting minor cortical bone changes, particularly in regions with thin bone or low contrast between structures. The resolution of CBCT and potential partial volume effects pose challenges in differentiating thin cortical bone from adjacent structures such as dentin. These limitations can lead to false negatives, underestimating or overlooking subtle cortical defects such as dehiscence or fenestration. Furthermore, reconstructed slice thickness, although not significantly impacting overall accuracy, might still affect the identification of fine bone details [49]. Another inherent limitation of this study is the subjective assessment of pain and swelling following surgery. However, our chosen method is currently the standard approach for evaluating pain and swelling within the endodontic literature. Developing a novel method for this purpose was beyond the scope of our study. The use of an established, widely adopted method facilitates comparison with existing research. Nonetheless, developing new methods to assess pain and swelling objectively and reduce subjective bias is certainly warranted in future research.

The results highlight the characteristics of the periapical lesions as a factor for predicting swelling after EMS. This study highlights the importance of using tailored approaches to minimize postoperative swelling in patients undergoing EMS with small periapical lesions and intact cortical bone. Utilizing advanced instrumentation, such as piezoelectric surgical handpieces, can reduce mechanical trauma and inflammation [66]. Integrating piezoelectric devices into dynamic navigation systems is proven to reduce the amount of bone cutting, thus reducing possible post-surgical edema, inflammation, and pain [67]. Furthermore, preoperative use of corticosteroids such as dexamethasone offers effective anti-inflammatory benefits [68]. Preoperative CBCT imaging enables risk stratification, allowing for individualized surgical and pharmacological interventions. By combining precise surgical techniques, pharmacological management, and patient education, these strategies aim to enhance recovery and optimize outcomes in EMS. Future research should consider larger sample sizes to enhance the reliability of the findings. Moreover, exploring the relationship between post-surgery pain and swelling and the success rates of endodontic microsurgery is an intriguing avenue for future research.

## 7. Conclusions

Within the limitations of this study, it can be concluded that patients who presented with periapical lesions larger than four millimeters in diameter were less likely to experience more swelling after EMS. More studies are needed to confirm the relationship between postoperative pain and swelling and the characteristics of the periapical lesion and cortical bone thickness.

## 8. Highlights

This study identified, for the first time, the association between post-surgical swelling and lesion size and cortical bone perforation. Patients presenting with large periapical lesions (periapical CBCT index score of >3) preoperatively are expected to develop less swelling after EMS. This study provides insights into predicting the severity of post-surgical pain and swelling to help clinicians take important steps to control them.

## Figures and Tables

**Table 1:** Baseline characteristics of patients (n = 61).

Study Variables	N
Age in years (mean ± SD)	39.8 ± 19.0
≤40 years	40 (65.6%)
≤40 years	21 (34.4%)
Gender	
Male	24 (39.3%)
Female	37 (60.7%)
Medical history	
Free	55 (90.2%)
Chronic illness	6 (9.8%)
Smoking	
Yes	9 (14.8%)
No	52 (85.2%)
Teeth count (mean ± SD)	1.36 ± 0.82
One	47 (77.0%)
More than one	14 (23.0%)
Tooth location *	
Anterior maxilla	20 (33.9%)
Posterior maxilla	22 (37.3%)
Anterior and posterior maxilla	2 (3.4%)
Anterior mandible	1 (1.7%)
Posterior mandible	14 (23.7%)
Anterior and posterior mandible	0
CBCT index *	
≤3	23 (39.0%)
>3	36 (61.0%)
CBCT cortical bone perforation *	
Expansion	5 (8.5%)
Destruction	36 (61.0%)
No expansion, no destruction	18 (30.5%)
Clinical presentation	
Symptomatic	32 (52.5%)
Asymptomatic	29 (47.5%)
Sinus tract	
Yes	11 (18.0%)
No	50 (82.0%)
Intraoperative cortical bone perforation	
Yes	33 (54.1%)
No	28 (45.9%)
Duration in minutes (mean ± SD)	131.6 ± 50.5
≤120 min	35 (57.4%)
>120 min	26 (42.6%)
Preoperative analgesics	
Yes	38 (62.3%)
No	23 (37.7%)
Preoperative mouthwash	
Yes	36 (59.0%)
No	25 (41.0%)
Postoperative mouthwash	
Yes	57 (93.4%)
No	4 (6.6%)
Postoperative antibiotics	
Yes	41 (67.2%)
No	20 (32.8%)
Postoperative analgesics	
Paracetamol and Ibuprofen	41 (67.2%)
No analgesics	1 (1.6%)
Ibuprofen only	19 (31.1%)

* Missing cases were excluded from the analysis.

**Table 2:** Descriptive statistics of pain and swelling measured over time (n = 61).

Parameters	Mean	SD	Median	Min	Max
Pain					
8 h	4.26	3.13	4.00	0	10
24 h	3.23	2.55	3.00	0	9
48 h	2.72	2.59	2.00	0	10
72 h	1.97	2.44	1.00	0	9
Overall mean pain score	3.04	2.09	3.00	0	8.25
Swelling					
8 h	4.07	2.91	4.00	0	10
24 h	6.46	2.87	7.00	0	10
48 h	6.11	2.79	7.00	0	10
72 h	4.36	2.77	4.00	0	10
Overall mean swelling score	5.25	2.18	5.50	0.5	9

**Table 3:** The relationship between overall pain and swelling among the baseline characteristics of patients (n = 61).

**Factor**	**Overall Pain Level**			**Overall Swelling Level**		
	**With Pain** **N%** **(n = 09)**	**No Pain** **N%** **(n = 52)**	***p*-Value ^§^**	**Swelling** **N%** **(n = 37)**	**No Swelling** **N%** **(n = 24)**	***p*-Value ^§^**
Age in years						
≤40 years	6 (66.7%)	34 (65.4%)	0.940	23 (62.2%)	17 (70.8%)	0.586
>40 years	3 (33.3%)	18 (34.6%)		14 (37.8%)	7 (29.2%)	
Gender						
Male	1 (11.1%)	23 (44.2%)	0.076	15 (40.5%)	9 (37.5%)	1.000
Female	8 (88.9%)	29 (55.8%)		22 (59.5%)	15 (62.5%)	
Medical history						
Free	8 (88.9%)	47 (90.4%)	1.000	33 (89.2%)	22 (91.7%)	1.000
Chronic illness	1 (11.1%)	5 (9.6%)		4 (10.8%)	2 (8.3%)	
Smoking						
Yes	2 (22.2%)	7 (13.5%)	0.609	7 (18.9%)	2 (8.3%)	0.462
No	7 (77.8%)	45 (86.5%)		30 (81.1%)	22 (91.7%)	
Teeth count						
One	7 (77.8%)	40 (76.9%)	1.000	30 (81.1%)	17 (70.8%)	0.370
More than one	2 (22.2%)	12 (23.1%)		7 (18.9%)	7 (29.2%)	
Tooth location *						
Maxilla	7 (77.8%)	37 (74.0%)	1.000	26 (74.3%)	18 (75.0%)	1.000
Mandible	2 (22.2%)	13 (26.0%)		9 (25.7%)	6 (25.0%)	
CBCT index						
≤3	4 (44.4%)	19 (38.0%)	0.725	18 (51.4%)	5 (20.8%)	0.029 **
>3	5 (55.6%)	31 (62.0%)		17 (48.6%)	19 (79.2%)	
CBCT cortical bone perforation *						
Expansion	1 (11.1%)	4 (8.0%)	0.751	3 (8.6%)	2 (8.3%)	0.841
Destruction	6 (66.7%)	30 (60.0%)		20 (57.1%)	16 (66.7%)	
None	2 (22.2%)	16 (32.0%)		12 (34.3%)	6 (25.0%)	
Clinical presentation						
Symptomatic	4 (44.4%)	28 (53.8%)	0.724	20 (54.1%)	12 (50.0%)	0.798
Asymptomatic	5 (55.6%)	24 (46.2%)		17 (45.9%)	12 (50.0%)	
Sinus tract						
Yes	3 (33.3%)	8 (15.4%)	0.343	6 (16.2%)	5 (20.8%)	0.738
No	6 (66.7%)	44 (84.6%)		31 (83.8%)	19 (79.2%)	
Intraoperative cortical bone perforation						
Yes	4 (44.4%)	29 (55.8%)	0.720	16 (43.2%)	17 (70.8%)	0.040 **
No	5 (55.6%)	23 (44.2%)		21 (56.8%)	7 (29.2%)	
Duration in minutes						
≤120 min	4 (44.4%)	31 (59.6%)	0.477	19 (51.4%)	16 (66.7%)	0.294
>120 min	5 (55.6%)	21 (40.4%)		18 (48.6%)	8 (33.3%)	
Preoperative analgesic						
Yes	7 (77.8%)	31 (59.6%)	0.462	23 (62.2%)	15 (62.5%)	1.000
No	2 (22.2%)	21 (40.4%)		14 (37.8%)	9 (37.5%)	
Preoperative mouthwash						
Yes	8 (88.9%)	28 (53.8%)	0.048 **	21 (56.8%)	15 (62.5%)	0.791
No	1 (11.1%)	24 (46.2%)		16 (43.2%)	9 (37.5%)	
Postoperative antibiotic						
Yes	5 (55.6%)	36 (69.2%)	0.458	25 (67.6%)	16 (66.7%)	1.000
No	4 (44.4%)	16 (30.8%)		12 (32.4%)	8 (33.3%)	
Postoperative mouthwash						
Yes	8 (88.9%)	49 (94.2%)	1.000	37 (100%)	20 (83.3%)	0.020 **
No	1 (11.1%)	3 (5.8%)		0	4 (16.7%)	
Postoperative Analgesics						
Paracetamol and Ibuprofen	8 (88.9%)	33 (63.5%)	360	24 (64.9%)	17 (70.8%)	0.382
No analgesics	0	1 (1.9%)		0	1 (4.2%)	
Ibuprofen only	1 (11.1%)	18 (34.6%)		13 (35.1%)	6 (25.0%)	

* Missing cases were excluded from the analysis. ^§^ *p*-value has been calculated using the Fischer Exact Test. ** Significant at *p* ≤ 0.05 level in bold.

**Table 4:** Multivariate regression analysis estimates to determine the significant independent factors associated with pain and swelling (n = 61).

Factor	Overall Pain			Overall Swelling		
	AOR	95% CI	*p*-Value ^§^	AOR	95% CI	*p*-Value ^§^
Intraoperative cortical bone perforation						
Yes	0.435	0.093–2.032	0.290	0.335	0.102–1.100	0.071
No	Ref			Ref		
Preoperative mouthwash						
Yes	0.712	0.154–3.292	0.662	0.412	0.125–1.356	0.145
No	Ref			Ref		
Postoperative mouthwash						
Yes	8.086	0.904–72.330	0.062	1.051	0.324–3.409	0.934
No	Ref			Ref		
CBCT index						
≤3	Ref			Ref		
>3	0.823	0.176–3.841	0.804	0.243	0.071–0.830	0.024 **

AOR—Adjusted Odds Ratio; CI—Confidence Interval. ^§^ *p*-value has been calculated using the Fischer Exact Test. ** Significant at *p* ≤ 0.05 level in bold.

## Data Availability

Data is contained within the article.
